# Evaluating early lymphocyte-to-monocyte ratio as a predictive biomarker for delirium in older adult patients with sepsis: insights from a retrospective cohort analysis

**DOI:** 10.3389/fmed.2024.1342568

**Published:** 2024-01-31

**Authors:** Xiaopeng Shi, Lei Yang, Weimin Bai, Lijuan Jing, Lijie Qin

**Affiliations:** Department of Emergency, Henan Provincial People’s Hospital, Zhengzhou University People’s Hospital, Zhengzhou, China

**Keywords:** lymphocyte-to-monocyte ratio, delirium, sepsis, older adult, predictive modeling, MIMIC-IV

## Abstract

**Background:**

This study aims to explore the value of the Lymphocyte-to-Monocyte Ratio (LMR) in predicting delirium among older adult patients with sepsis.

**Methods:**

Retrospective data were obtained from the MIMIC-IV database in accordance with the STROBE guidelines. Patients aged 65 and above, meeting the Sepsis 3.0 criteria, were selected for this study. Delirium was assessed using the Confusion Assessment Method for the ICU (CAM-ICU). Demographic information, comorbid conditions, severity of illness scores, vital sign measurements, and laboratory test results were meticulously extracted. The prognostic utility of the Lymphocyte-to-Monocyte Ratio (LMR) in predicting delirium was assessed through logistic regression models, which were carefully adjusted for potential confounding factors.

**Results:**

In the studied cohort of 32,971 sepsis patients, 2,327 were identified as meeting the inclusion criteria. The incidence of delirium within this subgroup was observed to be 55%. A univariate analysis revealed a statistically significant inverse correlation between the Lymphocyte-to-Monocyte Ratio (LMR) and the risk of delirium (*p* < 0.001). Subsequent multivariate analysis, which accounted for comorbidities and illness severity scores, substantiated the role of LMR as a significant predictive marker. An optimized model, achieving the lowest Akaike Information Criterion (AIC), incorporated 17 variables and continued to demonstrate LMR as a significant prognostic factor (*p* < 0.01). Analysis of the Receiver Operating Characteristic (ROC) curve indicated a significant enhancement in the Area Under the Curve (AUC) upon the inclusion of LMR (*p* = 0.035).

**Conclusion:**

The Lymphocyte-to-Monocyte Ratio (LMR) serves as a significant, independent prognostic indicator for the occurrence of delirium in older adult patients with sepsis. Integrating LMR into existing predictive models markedly improves the identification of patients at elevated risk, thereby informing and potentially guiding early intervention strategies.

## 1 Introduction

Sepsis, which manifests as life-threatening organ dysfunction due to a dysregulated host response to infection, annually impacts millions worldwide and is a principal cause of deteriorating global health (1). It has been established that advanced age correlates with an elevated risk of sepsis and an increased mortality rate among septic patients (2), with older adults accounting for over half of all severe sepsis incidents (3). Delirium, marked by an acute reduction in cognitive capabilities, constitutes a frequent, grave, and often lethal challenge, affecting as many as 50% of hospitalized older individuals (4). Evidence suggests that individuals aged 65 years or older are at heightened risk for sepsis-associated delirium (SAD), which is closely linked with the severity of their septic condition (5). Delirium not only commonly complicates the clinical picture for older adult patients with sepsis but also significantly contributes to extended hospital stays, increased fatality rates, and escalated healthcare expenditures (6). Moreover, the incidence of delirium bears a direct relation to the long-term prognosis of patients, with severe instances potentially culminating in enduring cognitive impairment or mortality.

Contemporary studies have established that the emergence of sepsis-associated delirium (SAD) is multifactorial, involving neuroinflammatory responses, microcirculatory impairments, metabolic anomalies, and neurotransmitter dysregulation (7). The prevailing diagnostic approach for SAD employs clinical tools such as the Confusion Assessment Method (CAM) and the Delirium Rating Scale (DRS) (8). These tools are designed to facilitate prompt delirium detection by clinicians, though their deployment in high-paced clinical environments may be constrained by requirements for specialized training and time commitment. Additionally, assessing patients with unstable levels of consciousness poses considerable difficulty. Research identifying delirium predictors has linked factors such as advanced age, pre-existing cognitive deficits, infection severity, and specific laboratory measures—including white blood cell count and serum creatinine levels—to the likelihood of delirium onset (9). Despite these insights, the field still faces a deficit of biomarkers that possess both high sensitivity and specificity for delirium prediction (10). The innovation and application of objective, precise assessment instruments and biomarkers, coupled with a deeper understanding of SAD’s pathophysiology, are imperative for enhancing the clinical prognosis of older adult patients with sepsis.

Recent investigations have brought the lymphocyte-to-monocyte ratio (LMR) to the fore as a biomarker with promising utility in indicating immune status. LMR is posited to forecast acute inflammatory responses and the advent of sepsis (11). It is posited that a balance between adaptive and innate immune responses is crucial in the etiology of sepsis-induced delirium (12). Employing retrospective data from the MIMIC-IV database, this study evaluated LMR’s predictive efficacy for delirium in septic senior patients. The aim was to ascertain LMR’s viability as a non-invasive, cost-efficient, and easily accessible biomarker for the precocious identification of delirium in this patient population.

## 2 Materials and methods

### 2.1 Data source

Open-source medical information was employed from the Medical Information Mart for Intensive Care (MIMIC-IV version 2.2) database for this investigation. The MIMIC-IV repository encompasses extensive, high-quality patient data spanning from 2008 to 2019, sourced from the Beth Israel Deaconess Medical Center (13). The database amalgamates diverse clinical datasets including case histories, pharmacotherapy records, laboratory findings, patient demographics, and diagnostic codes based on the International Classification of Diseases. The principal investigator (Xiaopeng Shi) successfully completed the requisite online training under the auspices of the National Institutes of Health’s collaborative institutional training initiative. Subsequent to this training, authorization was secured from the Institutional Review Board of the Massachusetts Institute of Technology for database access and data retrieval from MIMIC-IV (Certification number 38652558). Both the Massachusetts Institute of Technology (No. 0403000206) and the Beth Israel Deaconess Medical Center (2001-P-001699/14) provided ethical approval for the database’s research utilization. The manuscript conforms to the STROBE guidelines, which delineate standards for reporting observational studies (14).

### 2.2 Patients

The results of the Confusion Assessment Method for the Intensive Care Unit (CAM-ICU) assessments, used to evaluate the presence of delirium, are recorded in the MIMIC-IV database. This method includes criteria such as acute onset with marked fluctuations in consciousness, inattention, disorganized thinking, and acute changes in the level of consciousness. A positive delirium diagnosis was established if the criteria (1) + (2) + (3) or (1) + (2) + (4) were satisfied. Patients were subsequently stratified into either the delirium or non-delirium cohort, contingent upon any positive CAM-ICU assessment during their ICU tenure.

### 2.3 Study settings

Inclusion criteria were anchored to the 2016 Sepsis 3.0 definition and diagnostic benchmarks promulgated by the American Society of Critical Care Medicine and the European Society of Intensive Care Medicine, delineating Sepsis 3.0 as an infection conjoined with a Sequential Organ Failure Assessment (SOFA) score of 2 or higher (15). Only the data from the first ICU admission were considered for patients with multiple hospital stays.

The study excluded individuals under the age of 65, patients who either died or left the ICU within 48 h of admission, and those with primary diseases causing brain injury, such as ischemic or hemorrhagic stroke, psychiatric disorders, or dementia. Additionally, patients diagnosed with malignant tumors and those who did not undergo a delirium assessment were also excluded.

### 2.4 Data collection

Data were extracted from the MIMIC-IV database using structured query language with PostgreSQL. The following information was retrieved: (1) demographic data: age, sex, and height; (2) comorbidities: chronic pulmonary disease, diabetes, chronic liver disease, and chronic renal disease; (3) severity of illness scores on the first day in the ICU: Acute Physiology Score III (APSIII), Logistic Organ Dysfunction System (LODS), Oxford Acute Severity of Illness Score (OASIS), Glasgow Coma Scale (GCS), and Charlson Comorbidity Index (CCI); (4) vital signs on the first day of ICU admission: heart rate (HR), respiratory rate (RR), mean arterial pressure (MAP), temperature (T), mechanical ventilation status, and peripheral oxygen saturation (SpO2); (5) laboratory results on the first day of ICU admission: hemoglobin, platelet count, white blood cell count (WBC), albumin, anion gap, bicarbonate, blood urea nitrogen (BUN), calcium, sodium, potassium, international normalized ratio (INR), prothrombin time (PT), partial thromboplastin time (PTT), alanine aminotransferase (ALT), aspartate aminotransferase (AST), total bilirubin (TBIL), SpO2, glucose, and lactate.

### 2.5 Statistical analysis

The distribution of continuous variables was appraised using the Shapiro–Wilk test, revealing non-normality. Accordingly, such variables are delineated as medians with interquartile ranges (IQR), and were compared via the Mann–Whitney U test or Wilcoxon rank-sum test, contingent upon their suitability. Categorical variables are expressed as counts (percentages) and were subjected to the chi-square test or Fisher’s exact test, as dictated by the dataset’s characteristics.

In the univariate analysis phase, LMR served as the exclusive predictor in the logistic regression model’s construction. For the multivariate analysis, the models were sequentially enriched with additional variables: Model 1, the foundational model, incorporated solely LMR; Model 2 integrated various vital signs and comorbidities, refining Model 1; Model 3 appended laboratory results to the previously adjusted Model 2; and Model 4, adjusted for the elements in Model 3, included a variety of scoring metrics. To avert multicollinearity, neither monocytes nor lymphocytes were included in these models. Furthermore, a backward stepwise logistic regression approach (Model 5) was executed to procure the most parsimonious model, as indicated by the minimum Akaike Information Criterion (AIC) (16). The presence of collinearity among continuous variables was gauged using the variance inflation factor (VIF) (17), with any variable exhibiting a VIF exceeding 5 being excluded from the final model (18). Additionally, the relationship between LMR and delirium was graphically depicted using restricted cubic splines. The prognostic power of LMR for predicting the presence of delirium was quantified by calculating the sensitivity and specificity, and by constructing ROC curves for regression models, both inclusive and exclusive of LMR. The enhancement of predictive performance by LMR was measured by the area under the ROC curve (AUC), with the DeLong method applied to assess any significant shifts in AUC resultant from LMR inclusion. All statistical analyses were performed using R version 4.3.2, and *p* < 0.05 was considered statistically significant.

## 3 Results

### 3.1 Baseline characteristics

**Figure 1:**
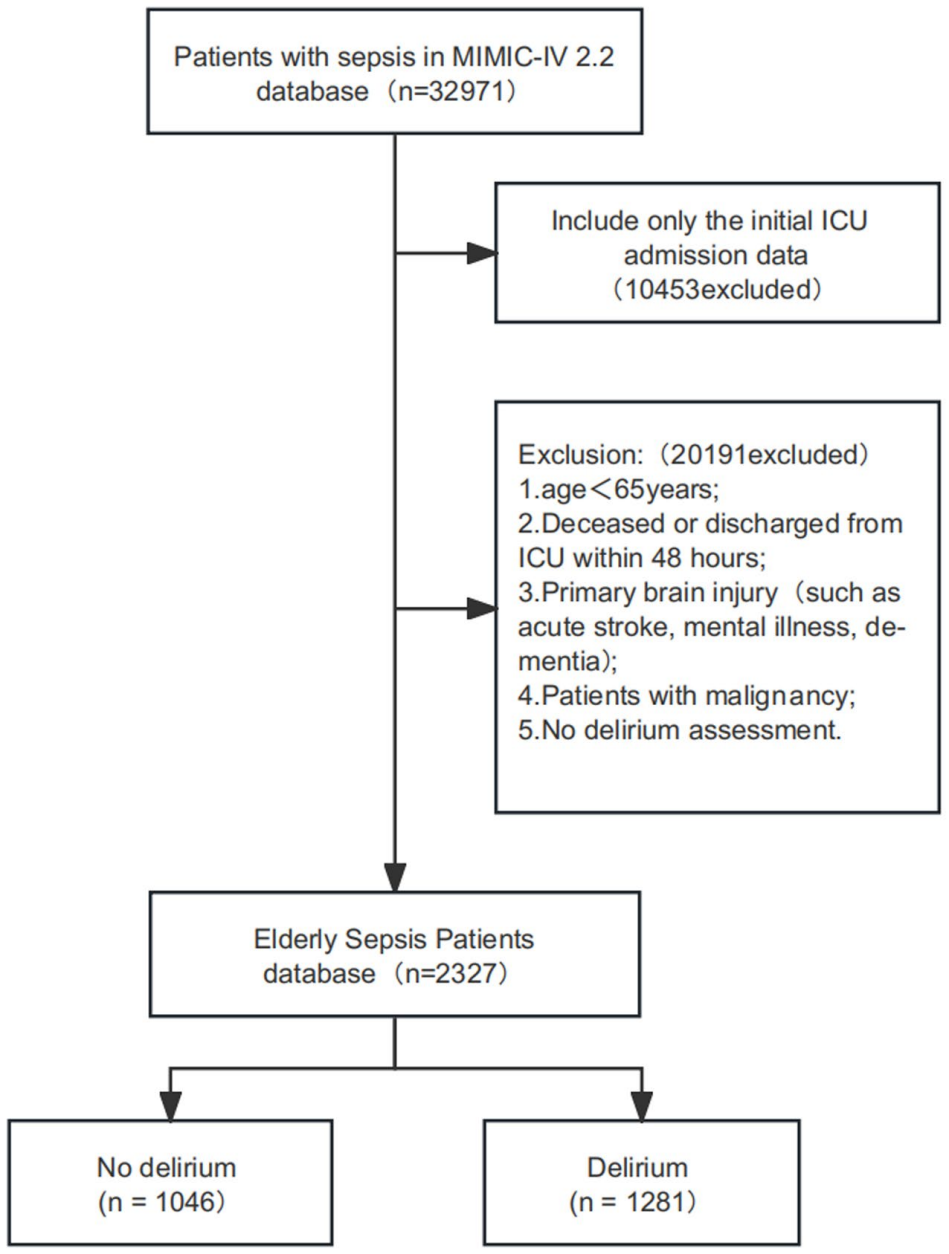
Flow chart for patient selection. ICU: intensive care unit; MIMIC-IV: medical information mart for intensive care IV.

A total of 32,971 sepsis patients were retrieved from the MIMIC IV 2.2 database. Based on the exclusion criteria, 30,644 patients were excluded. Ultimately, 2,327 older adult patients with sepsis were included in this study, as shown in Figure 1. The median age of the included patients was 76.71 years (IQR: 70.4–83.44), with 55% being male. There were 1,281 cases of delirium, resulting in a delirium incidence of 55% among the older adult patients with sepsis. Compared to patients without delirium, those with delirium had higher APSIII, LODS, and OASIS scores, as well as increased heart rate, respiratory rate, and temperature; a higher proportion required mechanical ventilation, and there were higher rates of chronic renal and liver failure, as well as a higher comorbidity index. Additionally, these patients exhibited increased white blood cell counts, higher anion gaps, and elevated levels of blood urea nitrogen, creatinine, monocytes, neutrophils, ALT, AST, TBIL, while bicarbonate, sodium, platelet counts, and lymphocytes were lower, along with a decreased GCS score and lower LMR, as detailed in Table 1.

**Table 1:** Baseline characteristics of the included patients.

Baseline variables	Total (*n* = 2327)	No delirium (*n* = 1046)	Delirium (*n* = 1281)	*p*-value
Age (years), median (Q1,Q3)	76.71 (70.4, 83.44)	76.62 (70.4, 83.78)	76.82 (70.38, 83.22)	0.955
Height(cm), Median (Q1,Q3)	169.76 (165, 173)	169.76 (165, 170)	169.76 (163, 173)	0.67
Male, n (%)	1288 (55)	568 (54)	720 (56)	0.38
Severe score (median [Q1,Q3])				
APSIII	49 (38, 63)	44 (35, 57)	54 (42, 67)	< 0.001
LODS	6 (4, 8)	5 (3, 7)	7 (5, 9)	< 0.001
OASIS	35 (30, 41)	33 (27, 38)	37 (32, 43)	< 0.001
GCS	15 (14, 15)	15 (14, 15)	15 (13, 15)	< 0.001
CCI	6 (4, 7)	5 (4, 7)	6 (4, 7)	< 0.001
Vital signs, median [IQR]				
HR(beats/minute)	83.33 (74.42, 95.14)	81.82 (74.13, 92.66)	84.52 (74.54, 97.24)	0.001
MAP (mmHg)	73.42 (68.31, 79.26)	73.13 (68.21, 78.29)	73.89 (68.42, 80.07)	0.021
RR (breath/minute)	19.2 (17.09, 22.15)	18.74 (16.76, 21.69)	19.59 (17.39, 22.4)	< 0.001
Temperature (°C)	36.85 (36.6, 37.06)	36.78 (36.57, 36.94)	36.91 (36.64, 37.15)	< 0.001
Ventilation, *n* (%)	1528 (66)	517 (49)	1011 (79)	< 0.001
Spo2 (%)	97.23 (95.66, 98.51)	97.18 (95.67, 98.43)	97.29 (95.66, 98.59)	0.223
Comorbidity, *n* (%)				
COPD	773 (33)	332 (32)	441 (34)	0.185
DM	837 (36)	364 (35)	473 (37)	0.308
CKD	708 (30)	295 (28)	413 (32)	0.039
CLD	84 (4)	25 (2)	59 (5)	0.006
Laboratory parameters (median [Q1,Q3])				
Hemoglobin (g/dL)	9.5 (8.1, 11.05)	9.5 (8.1, 10.9)	9.6 (8.1, 11.2)	0.135
Platelet (K/uL)	152 (109, 211.5)	145.5 (108, 203)	157 (109, 220)	0.012
WBC (K/uL)	15.1 (11.2, 20.2)	14.9 (10.8, 19.6)	15.5 (11.4, 20.4)	0.019
Albumin (g/dL)	3.06 (3, 3.1)	3.06 (3.06, 3.06)	3.06 (2.9, 3.2)	0.303
Aniongapp (mEq/L)	17 (14, 20)	16 (14, 19)	18 (15, 21)	< 0.001
Bicarbonate (mEq/L)	24 (21, 26)	24 (22, 26)	23 (21, 26)	0.014
BUN (mg/dL)	28 (19, 46)	26 (17.25, 40)	31 (20, 51)	< 0.001
Calcium (mg/dL)	8.51 (8.1, 8.9)	8.5 (8.1, 8.88)	8.51 (8.1, 9)	0.031
Creatinine (mg/dL)	1.3 (0.9, 2.1)	1.2 (0.9, 1.8)	1.4 (1, 2.3)	< 0.001
Sodium (mEq/L)	140 (137, 143)	140 (138, 143)	140 (137, 142)	< 0.001
Potassium (mEq/L)	4.6 (4.2, 5.1)	4.6 (4.2, 5)	4.6 (4.2, 5.2)	0.004
Monocytes (K/uL)	0.62 (0.38, 0.97)	0.57 (0.35, 0.86)	0.67 (0.4, 1.08)	< 0.001
Lymphocytes (K/uL)	1.15 (0.72, 1.74)	1.24 (0.77, 1.93)	1.09 (0.68, 1.61)	< 0.001
Neutrophils (K/uL)	10.56 (7.34, 14.97)	10.07 (7.11, 14.24)	10.93 (7.51, 15.71)	< 0.001
INR	1.4 (1.2, 1.76)	1.45 (1.2, 1.7)	1.4 (1.2, 1.8)	0.781
PT (sec)	15.7 (13.3, 19.1)	15.8 (13.62, 18.88)	15.5 (13.1, 19.9)	0.58
PTT (sec)	35.6 (29.7, 49.95)	35.4 (29.8, 49.45)	35.8 (29.6, 50.4)	0.895
ALT (U/L)	84 (23, 290.34)	210 (27, 290.34)	52 (21, 290.34)	< 0.001
AST (U/L)	137 (35, 469.08)	294.5 (42.25, 469.08)	92 (33, 469.08)	< 0.001
TBIL (mg/dL)	1.5 (0.6, 2.36)	2.36 (0.7, 2.36)	1.1 (0.5, 2.36)	< 0.001
Glucose (mg/dL)	136 (118.05, 166.23)	132.65 (117.83, 155.81)	139.5 (118.4, 173.75)	< 0.001
NLR	9.13 (5.14, 16.02)	7.92 (4.67, 13.92)	10.13 (5.8, 17.6)	< 0.001
PLR	176 (103.96, 302.44)	152.77 (94.02, 274.31)	195.45 (117.43, 326.67)	< 0.001
LMR	1.83 (1, 3.32)	2.25 (1.18, 3.95)	1.57 (0.9, 2.87)	< 0.001
Lactate (mmol/L)	3.1 (1.8, 3.4)	3.2 (1.9, 3.4)	3 (1.8, 3.8)	0.887

SOFA: sequential organ failure assessment; APSIII: acute physiology score III; LODS: logistic organ dysfunction system; OASIS: oxford acute severity of illness score; GCS: Glasgow coma scale; CCI: Charlson comorbidity index DM: diabetes mellitus; COPD: chronic obstructive pulmonary diseases; CKD: chronic kidney disease; CLD: chronic liver disease; HR: heart rate; RR: respiratory rate; MAP: mean arterial pressure; BUN: blood urea nitrogen; WBC: white blood cell count; INR: international normalized ratio; PT: prothrombin time; PTT: partial thromboplastin time; ALT: alanine aminotransferase; AST: aspartate aminotransferase; TBIL: total bilirubin; NLR: neutrophil to lymphocyte ratio; PLR: platelet to lymphocyte ratio; LMR: lymphocyte to monocyte ratio.

### 3.2 Association between LMR and delirium incidence

Univariate logistic regression analysis (Model 1) revealed that each incremental increase in LMR was inversely correlated with the likelihood of delirium onset, evidenced by a decrease of 0.12845 in the log-odds ratio, a result bearing statistical significance (*p* < 0.001). Subsequent multivariate logistic regression models (Models 2, 3, and 4) upheld the significance of LMR as a predictive factor, even when accounting for the influence of additional variables. In the culminating model (optimal_model), a unit augmentation in LMR was significantly associated with a reduction of 0.09911 in the log-odds ratio for the occurrence of delirium (*p* < 0.001). Additional variables, inclusive of HR, MAP, body temperature, CLD, platelet count, WBC, anion gap, bicarbonate, BUN, sodium, PTT, TBIL, SpO2, and scores from SOFA, LODS, OASIS, and GCS, were assimilated into the final model. While a robust negative association between LMR and delirium was discernible in the foundational model, the introduction of further variables led to a progressive elevation of the OR, indicating a diluted impact of LMR on delirium manifestations. Nevertheless, as model complexity intensified, the correlation between LMR and delirium attenuated, with the OR converging towards unity (Table 2).

**Table 2:** The correlation between lymphocyte-to-monocyte ratio (LMR) and the occurrence of delirium in older adult patients with sepsis.

	Univariable analysis model 1	Multivariable analysis model 2	Multivariable analysis model 3	Multivariable analysis model 4
OR (95% CI)	0.8795 (0.8495, 0.9095)	0.8991 (0.8678, 0.9305)	0.9033 (0.8696, 0.9374)	0.9104 (0.8751, 0.9462)
*p*-value	*p* < 0.001	*p* < 0.001	*p* < 0.001	*p* < 0.001

Utilizing backward stepwise logistic regression, a model was identified with the minimum Akaike Information Criterion (AIC) value, concluding at 2853. This model was constructed by integrating 17 variables. Among these variables, it was found that LMR (OR = 0.9056, *p* < 0.01), heart rate (HR, OR = 0.9927, *p* = 0.0218), mean arterial pressure (MAP, OR = 1.0273, *p* < 0.01), body temperature (OR = 1.6826, *p* < 0.01), chronic liver disease (CLD, OR = 1.7491, *p* = 0.0401), anion gap (OR = 1.0346, *p* = 0.0026), sodium levels (OR = 1.034, *p* = 0.001), total bilirubin (TBIL, OR = 0.961, *p* = 0.0398), Sequential Organ Failure Assessment score (SOFA, OR = 1.0559, *p* = 0.0401), Logistic Organ Dysfunction Score (LODS, OR = 1.1872, *p* < 0.01), Oxford Acute Severity of Illness Score (OASIS, OR = 1.0451, *p* < 0.01), and Glasgow Coma Scale score (GCS, OR = 1.0635, *p* = 0.001) exhibited significant independent correlations with an elevated risk of delirium onset (Table 3).

**Table 3:** Variables selected through stepwise logistic regression analysis.

	OR	95%CI	*p*-value
LMR	0.9056	0.8712–0.9406	<0.01
HR	0.9927	0.9865–0.9989	0.0218
MAP	1.0273	1.0163–1.0387	<0.01
Temperature	1.6826	1.4182–2.0050	<0.01
CLD	1.7491	1.0368–3.0284	0.0401
Platelet	1.00096	0.99998–1.00196	0.0566
WBC	0.9921	0.9817–1.0025	0.1357
Aniongap	1.0346	1.0122–1.0580	0.0026
Bicarbonate	1.0185	0.9969–1.0407	0.0952
BUN	0.9966	0.9924–1.0009	0.1246
Sodium	1.034	1.0136–1.0550	0.001
PTT	0.9973	0.9946–1.0001	0.0569
TBIL	0.961	0.9215–0.9956	0.0398
Spo2	1.054	1.0088–1.1014	0.0189
SOFA	1.0559	1.0027–1.1125	0.0401
LODS	1.1872	1.1318–1.2461	<0.01
OASIS	1.0451	1.0304–1.0602	<0.01
GCS	1.0635	1.0250–1.1034	0.001

**Figure 2:**
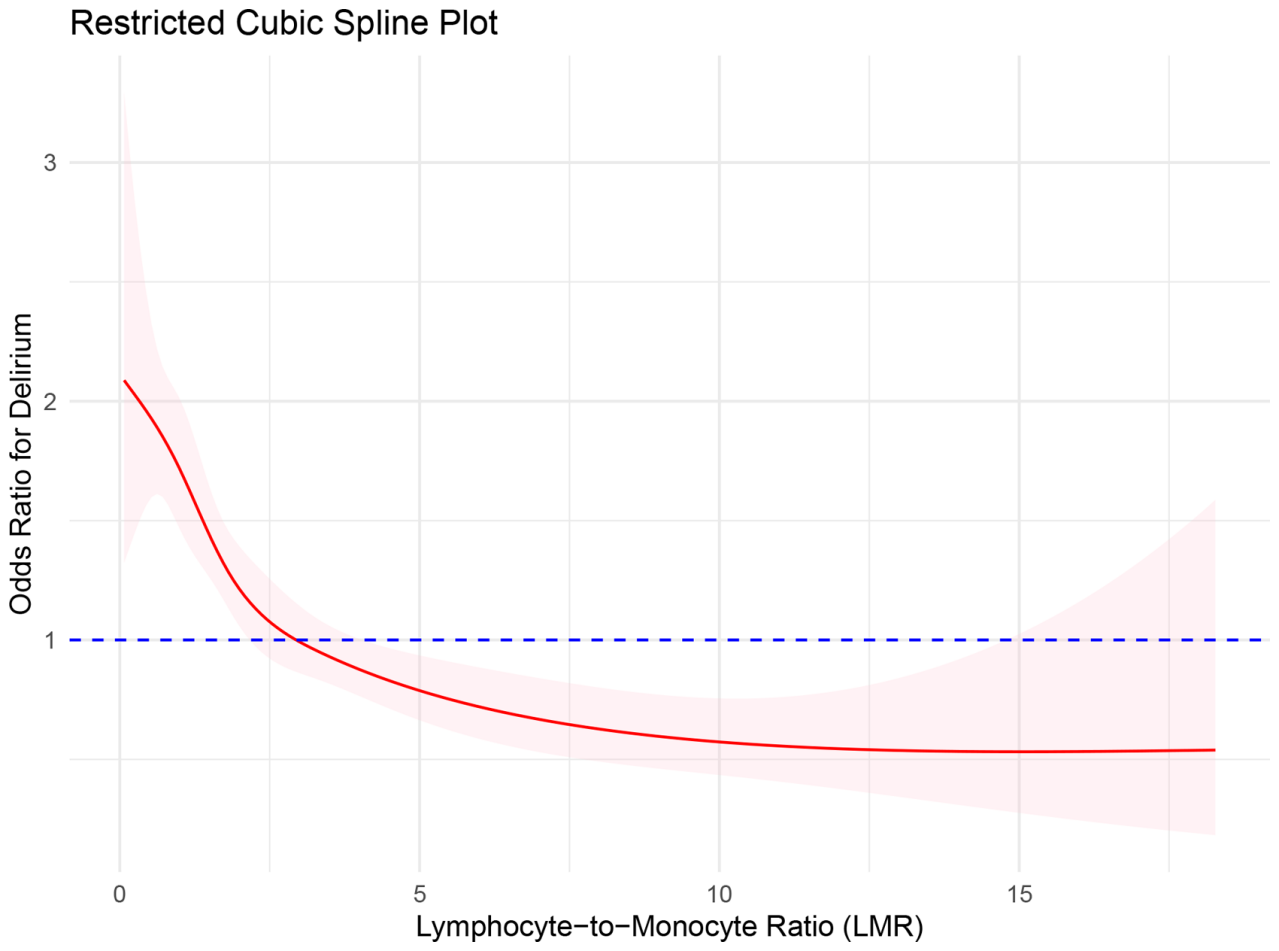
Restricted spline curve for the relationship between LMR and delirium in older adult patients with sepsis. The red bold line represents the odds ratio, and the shaded area represents the 95% confidence interval.

The analysis utilized restricted cubic splines to characterize the preoperative LMR’s association with delirium incidence. As demonstrated in Figure 2, the relationship between LMR and the odds ratio (OR) for delirium was found to be non-linear. When LMR was lower, there was a correspondingly higher OR, which sharply decreased with an increase in LMR, indicating an inverse correlation between LMR and delirium risk. However, at higher levels of LMR, the OR for delirium began to ascend slowly, potentially indicating that an excessively elevated LMR is associated with an augmented risk of delirium.

**Figure 3:**
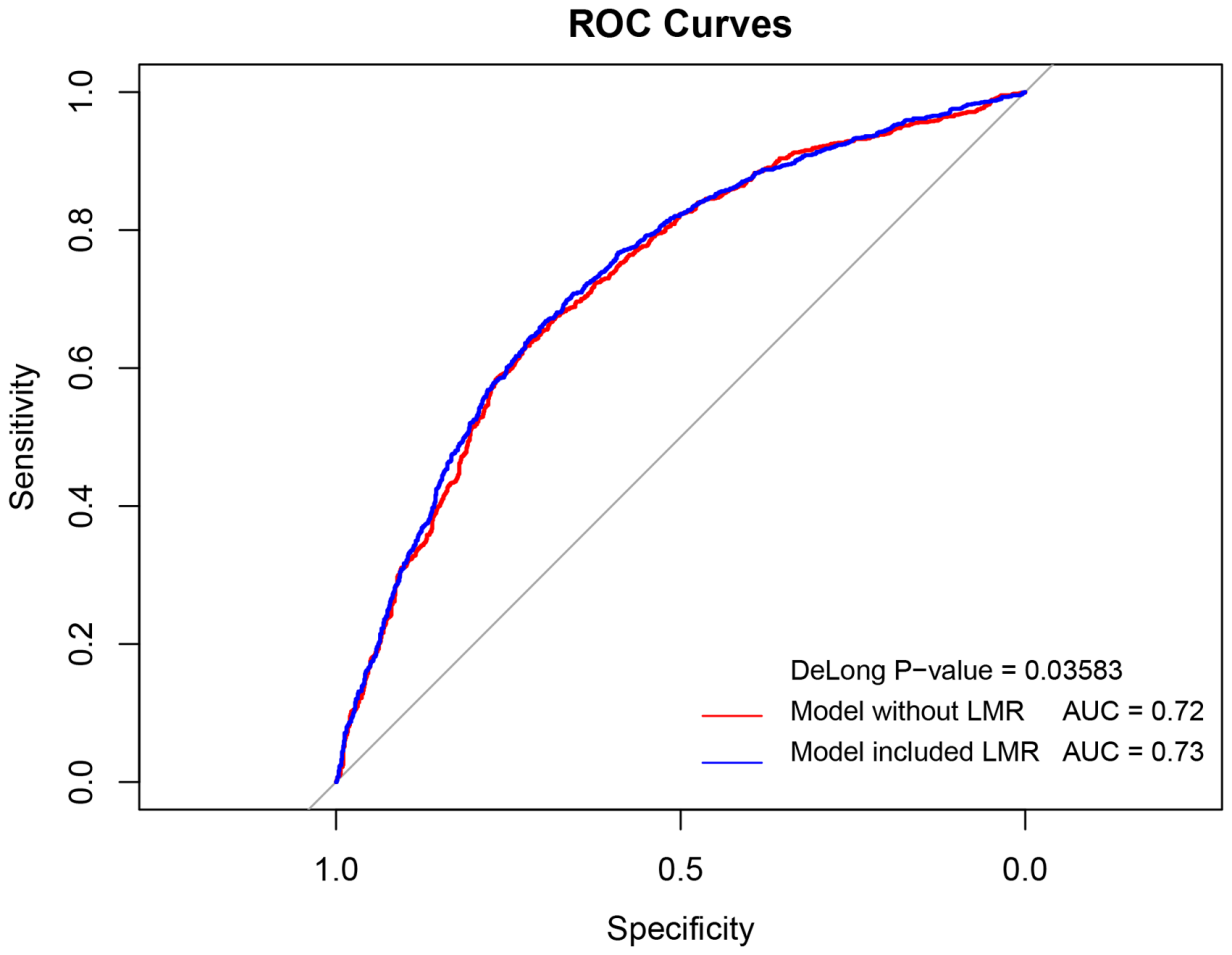
ROC curves for Multivariate Model Predictions of delirium in older adult patients with sepsis with and without LMR. ROC: receiver operating characteristic curve; AUC: area under the receiver operating characteristic curve; OR: odds ratio.

Receiver Operating Characteristic (ROC) curves were generated within the optimal model framework to evaluate the prognostic utility for delirium, both with the inclusion of the Lymphocyte-to-Monocyte Ratio (LMR) and without. The area under each curve (AUC) served as a quantitative measure of each model’s predictive efficacy. The integration of LMR into the model was associated with a marginal enhancement in AUC, and the comparative analysis of the ROC curves demonstrated statistical significance (DeLong, *p* = 0.035), as depicted in Figure 3.

## 4 Discussion

This study conducted an in-depth analysis of the delirium incidence within the older adult patients with sepsis population. Our data reveals a 55% incidence of delirium among these patients, in contrast to the 17.7 to 48% range reported in intensive care units (ICU) for sepsis-associated delirium (SAD) (9), with some studies indicating rates up to 70% (19). Additionally, another multicenter study documented acute mental status changes in 307 of 1,333 patients with severe sepsis (23%) (20). The variation in reported incidence rates may stem from differences in patient demographics, severity of sepsis, delirium diagnostic criteria, and methods of data collection and analysis. Our findings highlight the clinical importance of delirium in older adult patients with sepsis and identify multiple biomarkers and clinical parameters associated with an increased incidence of delirium, suggesting the need for heightened vigilance and comprehensive assessment in this vulnerable group to mitigate the impact of delirium on morbidity and mortality.

The mechanisms through which sepsis incites delirium are not fully understood, yet current research implicates a complex interplay of neuroinflammation, impaired cerebral perfusion, blood–brain barrier compromise, and neurotransmitter transport dysfunction. It has been observed that sepsis-induced endothelial cell activation escalates systemic inflammatory markers such as IL-1β, IL-6, and TNF-α, as well as the production of reactive oxygen species, factors that may accelerate the onset of sepsis-associated delirium (SAD) (12). Notably, the criteria for defining and applying SAD risk factors vary across studies. Ely et al. (21) found that over half of non-sedated septic patients exhibited SAD, correlating with bacteremia, and elevated levels of blood urea nitrogen and bilirubin. Ai Yuhang et al. (22) noted SAD in a subset of ICU patients, marked by heightened APACHE II scores. In contrast, Pandharipande et al. recognized SAD in a majority of their study cohort, with age and functional dependency as contributing risks (5). The single-center nature of these studies, however, introduces limitations such as small sample sizes and inadequate consideration of ICU-specific risk factors. Ebersoldt et al.’s multicenter research corroborated advanced age as an independent risk factor for SAD (23). These factors, while potentially central to SAD’s pathophysiology, await further investigation to establish causality (24).

In this study, we observed a significant inverse correlation between the lymphocyte-to-monocyte ratio (LMR) and the risk of delirium. This finding, not widely reported in the literature, suggests that LMR could serve as a potential biomarker for predicting and monitoring delirium risk in older adult patients with sepsis. Our multivariate logistic regression model further substantiated the significant association between increased LMR and reduced delirium risk, even after adjusting for other known risk factors. While aligning with literature on the close link between delirium and poor outcomes in sepsis patients, our data suggests that LMR may possess greater predictive value. This could be attributed to the use of an updated database, a broader patient cohort, and a more sophisticated statistical model. Hence, our findings underscore the potential significance of LMR as a novel biomarker for predicting delirium in sepsis and its potential direct impact on clinical management of sepsis patients.

The Confusion Assessment Method for the Intensive Care Unit (CAM-ICU) is the most widely utilized tool, designed specifically for the ICU setting and proven to be highly specific in assessing delirium among critically ill patients. However, significant heterogeneity in CAM-ICU results across different study settings has been noted, with potential low sensitivity in real-world clinical practice (25). The incidence of delirium varies greatly among ICU patient populations, possibly due to predisposing risk factors such as age and depression (26). The CAM-ICU faces challenges in assessing patients with fluctuating consciousness levels and in the absence of definitive diagnostic criteria for SAD. In this scenario, the LMR emerges as an objective biomarker, offering a novel approach. Its utility is underscored by its independence from subjective patient responses and variations in consciousness, rendering it particularly beneficial for patients who are unsuitable for traditional delirium assessments. Moreover, LMR’s ability to reflect the body’s inflammatory and immune status is potentially closely linked to the pathophysiological mechanisms involved in mental disorder development. This connection is crucial, given the established association between delirium and disruptions in inflammatory and immune responses. Consequently, LMR acts as a pivotal indicator of a patient’s risk of delirium. Its proficiency in mirroring these systemic states provides vital insights into the likelihood of a patient being at high risk for SAD, thereby aiding in the prompt identification and intervention. These characteristics elevate LMR as an indispensable tool in delirium assessment, significantly enhancing the capacity for early detection and effective management of patients predisposed to this intricate condition.

The development of delirium is a multifaceted and complex process influenced by a myriad of biological and environmental factors. The lymphocyte-to-monocyte ratio (LMR), while showing promise as a predictive tool for delirium in older adult patients with sepsis, requires further empirical validation to ascertain its efficacy and reliability comprehensively. This need for additional substantiation is crucial, considering the multifactorial nature of delirium. Moreover, LMR’s effectiveness may vary across different clinical scenarios due to factors such as inflammation levels, comorbidities, and treatment approaches, which can significantly impact its predictive accuracy. Therefore, future research should concentrate on integrating LMR with other biomarkers to achieve a more holistic understanding of delirium’s pathophysiology. Such integration aims to refine the assessment and management of delirium, potentially leading to enhanced patient outcomes, particularly in complex cases influenced by interrelated factors. In summary, while LMR is a valuable indicator for predicting delirium, its application should be cautiously considered alongside other clinical assessments, acknowledging its limitations and the necessity for further research to confirm its clinical utility.

## 5 Conclusion

In the cohort of older adult patients with sepsis, this study has identified the lymphocyte-to-monocyte ratio (LMR) as a substantial independent predictor of delirium. Retrospective analyses have uncovered a significant inverse relationship between LMR and the risk of delirium, where each increment in LMR correlates with a reduced risk. The integration of LMR into multivariate predictive models markedly improves the detection of patients with a heightened risk for delirium, thus potentially informing early interventions. The findings advocate for the clinical utility of LMR in refining delirium risk assessment and management strategies in the geriatric sepsis population, warranting further investigation into its broader clinical efficacy.

## Data availability statement

The datasets presented in this study can be found in online repositories. The names of the repository/repositories and accession number(s) can be found in the article/supplementary material.

## Ethics statement

The studies involving humans were approved by the institutional review boards of the Massachusetts Institute of Technology and Beth Israel Deaconess Medical Center. The studies were conducted in accordance with the local legislation and institutional requirements. Written informed consent for participation was not required from the participants or the participants’ legal guardians/next of kin because the requirement for individual patient consent was waived because the project does not impact clinical care and all patient confidential information was anonymized, therefore, the patient’s consent is not required. All methods were carried out by relevant guidelines and regulations. Written informed consent was not obtained from the individual(s) for the publication of any potentially identifiable images or data included in this article because the institutional review boards of the Massachusetts Institute of Technology and Beth Israel Deaconess Medical Center. The requirement for individual patient consent was waived because the project does not impact clinical care and all patient confidential information was anonymized, therefore, the patient’s consent is not required. All methods were carried out by relevant guidelines and regulations.

## Author contributions

XS: Data curation, Methodology, Writing – original draft. LY: Methodology, Visualization, Writing – review & editing. WB: Software, Validation, Writing – review & editing. LJ: Formal analysis, Software, Writing – review & editing. LQ: Project administration, Writing – review & editing.
